# Molecular Insights into the Positive Role of Soybean Nodulation by GmWRKY17

**DOI:** 10.3390/ijms26072965

**Published:** 2025-03-25

**Authors:** Xiaorui Zhao, Chunhai Mai, Lintao Xia, Gaiya Jia, Xinhui Li, Yichu Lu, Zhenying Li, Hongbin Yang, Lixiang Wang

**Affiliations:** Houji Laboratory in Shanxi Province, College of Agriculture, Shanxi Agricultural University, Taiyuan 030031, China; zhaoxiaorui@sxau.edu.cn (X.Z.); maichunhai@sxau.edu.cn (C.M.); xialintao@sxau.edu.cn (L.X.); jiagaiya@sxau.edu.cn (G.J.); lixinhui@sxau.edu.cn (X.L.); luyichu1@sxau.edu.cn (Y.L.); lizhenying@sxau.edu.cn (Z.L.); yanghongbin@sxau.edu.cn (H.Y.)

**Keywords:** symbiotic nitrogen fixation, *GmWRKY17*, root nodule, CRISPR/Cas9, nod factor signaling pathway

## Abstract

Soybean is an important economic oilseed crop, being rich in protein and plant oil, it is widely cultivated around the world. Soybeans have been shown to establish a symbiotic nitrogen fixation (SNF) with their compatible rhizobia, resulting in the formation of nodules. Previous studies have demonstrated the critical roles of phytohormones, such as abscisic acid and cytokinin, in the process of legume nodulation. The present study investigated the role of GmWRKY17, a homolog of *Rosa hybrida* (*Rh*)*WRKY13* in regulating plant immunity through cytokinin content and abscisic acid signaling in soybean nodulation. Utilizing real-time PCR and histochemical staining, we demonstrated that *GmWRKY17* is predominantly expressed in soybean root nodules. Subsequently, we analyzed the function of *GmWRKY17*-overexpression, RNA interference (RNAi), and the CRISPR/Cas9 system. Overexpression of *GmWRKY17* significantly increases soybean nodule number, while RNAi or CRISPR/Cas9-mediated knockout of *GmWRKY17* resulted in a dramatic repression of nodule formation in soybeans. These results highlight that GmWRKY17 functions as a positive regulator involved in soybean nodulation. Furthermore, manipulation of *GmWRKY17* expression impacts the expression of genes associated with the nod factor (NF) signaling pathway, thereby influencing soybean nodulation. This study demonstrated that WRKY-type transcription factors are involved in the regulation of legume nodulation, offering new light on the molecular basis of the symbiotic interaction between legumes and rhizobia.

## 1. Introduction

The soybean (*Glycine max*) is one of the most important crops in the world, representing 50% of the global crop legume area and 68% of global production [1]. Nitrogen (N) is a critical macronutrient that regulates plant growth and development. Therefore, enhancing nitrogen use efficiency is essential for achieving high and stable crop yields [2]. In conditions where nitrogen is scarce, leguminous plants establish symbiotic root nodules with rhizobia, thereby facilitating biological nitrogen fixation to meet their substantial nitrogen requirements [3]. It is estimated that symbiotic nitrogen fixation (SNF) contributes approximately 50–70 teragrams of fixed nitrogen to support agricultural systems on an annual basis [1]. This efficient and environmentally sustainable strategy effectively addresses plant growth requirements under low-nitrogen conditions while significantly stimulating atmospheric nitrogen cycling. Consequently, SNF is considered one of the most critical approaches for acquiring bioavailable nitrogen in Earth’s ecosystems [1]. SNF is the result of a complex set of chemical and physical interactions between legumes and compatible rhizobia, including signaling processes that regulate gene expression, determine reciprocal partner selection, and inhibit plant defense responses. These signals also provide a pathway for bacteria to enter plant epidermis and cortical cells, induce root cell division and nodular meristem formation, and eventually give rise to thousands of specialized organelles called “symbiosomes,” each containing one or more nitrogen-fixing bacteroids [4].

The interaction between leguminous and rhizobia originates from a complex molecular signaling process. A substantial body of research has been dedicated to leguminous species, including *Lotus japonicus*, *Medicago truncatula*, and soybean, which have been shown to undergo three distinct stages: the initial recognition of symbiotic signals, the subsequent infection of rhizobia, and the resulting nodule development [5]. The process of nodulation and SNF in legumes is an adaptive response to environments with limited nitrogen availability. In such low nitrogen conditions, legumes secrete flavonoids into the rhizosphere, which are subsequently recognized by rhizobia present in the surrounding soil. This recognition triggers the accumulation of rhizobia at the root epidermis and root hairs, while the flavonoids also induce the expression of nodulation-related genes in rhizobia, culminating in the synthesis of specific lipo-chitin nodulation factors (Nod Factors, NFs) [6,7]. These nodulation factors are perceived by the NF receptors (NFRs), specifically LjNFR1/NFR5 and MtLYK3/NFP, which are located on the plasma membrane of root hair cells in leguminous plants. This perception initiates the nodulation signaling pathway [8,9,10,11]. The activation of NFRs has been shown to trigger membrane depolarization and significant fluctuations in intracellular calcium ion concentrations, as well as the generation of calcium peaks [12]. The calcium/calmodulin-dependent protein kinase, calcium-calmodulin kinase (CCaMK) located in the nucleus, decodes these oscillating calcium signals and interacts with its downstream target, CYCLOPS, and phosphorylates it. The phosphorylated transcription factor CYCLOPS subsequently enhances the transcription of downstream genes nodulation signaling pathways 1 and 2 (*NSP1* and *NSP2*) [13]. TFs NSP1 and NSP2, in conjunction with the pivotal regulatory DELLA proteins of gibberellin, form a substantial complex that further regulates the expression of essential nodulation genes in leguminous plants, including nodule inception (*NIN*) and *ERN1* [14,15]. The transcription factor NIN subsequently activates the transcription of downstream nodulation genes, thereby initiating the nodulation process. NIN is thus a crucial regulatory factor in the early stages of nodule development. *NIN* is expressed in the epidermis shortly following rhizobial infection, whereas its expression is sustained in the meristematic zone over a longer period, thereby promoting cell division [16,17,18,19].

Plant hormones are increasingly recognized as crucial regulatory factors in rhizobial symbiosis. The local accumulation of cytokines, strigolactones (SLs), and auxins has been demonstrated to promote nodule development, whereas ethylene, jasmonic acid (JA), abscisic acid (ABA), and gibberellins (GAs) have been shown to negatively regulate the formation of infection threads, thereby inhibiting root hair infection and nodule development [20]. However, the role of salicylic acid (SA) and brassinosteroids (BRs) in determinate and indeterminate nodules remains to be elucidated. Furthermore, there are interactive regulatory effects among plant hormones; for instance, cytokines and auxins can promote ethylene synthesis, which in turn activates plant immune responses and suppresses nodule formation and development [21]. Cytokinins in soybean also participate in the auto-regulation of nodulation (AON). The B-type response regulator GmRR11d has been shown to induce a hypersensitive response of soybean roots to cytokines, thereby activating the AON pathway. Concurrently, *GmRR11d* inhibits the expression of key genes in the nodule signaling pathway, further controlling the nodule number [22].

TFs such as NSP1, NSP2, CYCLOPS, NIN, and ERN are increasingly recognized as crucial regulatory factors in rhizobial symbiosis [23,24,25,26]. WRKY TFs are among the most extensive and distinct protein superfamilies, exclusively existing in higher plant species, first identified in sweet potato [27]. The WRKY superfamily is uniquely marked by an N-terminal WRKY domain, which contains the conserved heptapeptide sequence WRKYGQK, underscoring its evolutionary conservation. It has been demonstrated that mutations within this sequence significantly reduce its DNA-binding activity. Furthermore, a unique zinc finger structure is located at the C-terminal [28].

WRKY TFs exhibit specific binding to the W-box (TTGACT/C), a cis-regulatory element located in the promoter regions of target genes, with TGAC representing the core motif of the W-box [29]. WRKY TFs are known to regulate multiple developmental processes in plants, such as stem elongation, pollen development, leaf senescence, and seed maturation [30]. Genome-wide analysis has revealed the presence of 176 WRKY genes in the soybean genome [31]. Overexpression of *GmWRKY54* has been shown to enhance soybean tolerance to salt and drought, while *GmWRKY13* has been demonstrated to negatively regulate responses to salt stress and drought in soybean [32]. Furthermore, *GmWRKY2* enhances salt and drought tolerance through an interaction with *GmMYB174*, leading to suppression of *GmNAC29* expression. [33]. Overexpression of *GmWRKY12* has been shown to improve soybean tolerance to drought and salt stress [34]. Overexpression of *GmWRKY17* improves the drought tolerance of soybean [35]. In addition, *GmWRKY16* has been demonstrated to enhance *Arabidopsis* tolerance to drought and salt stress through ABA-mediated pathways [36].

Furthermore, WRKY TFs play a pivotal role in regulating plant–microbe interactions by controlling the expression levels of downstream genes [36,37]. However, the specific functions and molecular mechanisms of WRKY TFs in nitrogen fixation within leguminous plants remain poorly understood. Plant hormones, including cytokinin (CK) and ABA, have been shown to play critical and often opposing roles in plant growth, development, and responses to both abiotic and biotic stressors [38]. For instance, The *Rosa hybrida* transcription factor RhWRKY13 enhances protection against *Botrytis cinerea* by modulating CK levels and ABA responses [39]. It is well established that both hormones, CK and ABA, influence the formation and development of root nodules in legumes [40,41,42]. However, the crosstalk mechanism of CK and ABA in legume nodulation remains unknown. In this study, we identified GmWRKY17, a homolog of AtWRKY40, by phylogenetic analysis, as being associated with nodule formation in soybean. *GmWRKY17* expression is triggered by rhizobial infection and was mainly expressed in root nodules. *GmWRKY17* was then cloned. Furthermore, our findings reveal that the novel transcription factor GmWRKY17 promotes nodulation. Building on previous reports that GmWRKY17 enhances soybean drought tolerance, our study identifies a new genetic resource for improving symbiotic nitrogen fixation efficiency and sustainability in drought conditions. This research will contribute to a comprehensive understanding of the genetic basis underlying drought tolerance in symbiotic nodulation and nitrogen fixation, facilitating advanced genetic improvements in soybeans to increase the efficiency of symbiotic nitrogen fixation and grain yield under drought conditions.

## 2. Results

### 2.1. Multiple Sequence Alignment and Phylogenetic Analysis of GmWRKY17

Bioinformatics analysis revealed that GmWRKY17 is located on chromosome 6, with a full length of 1776 bp, and containing three introns (Figure S1A). CDD domain analysis indicated that GmWRKY17 encodes a WRKY domain spanning amino acids from 137 to 197 (Figure S1B). Prediction of the protein’s secondary structure demonstrated that GmWRKY17 is primarily composed of random coils (60.20%), α-helices (30.61%), and extended strands (9.18%) (Figure S1C). Phosphorylation site prediction analysis identified a total of 41 putative phosphorylation sites on *GmWRKY17*, including serine (Ser), threonine (Thr), and tyrosine (Tyr) phosphorylation sites (Figure S1D). Furthermore, hydropathy prediction for *GmWRKY17* indicated that the number of hydrophilic amino acids (hydrophilic index < 0) was significantly higher than the number of hydrophobic amino acids (hydrophilic index > 0), suggesting that this protein may be classified as a hydrophilic protein (Figure S1E).

**Figure 1:**
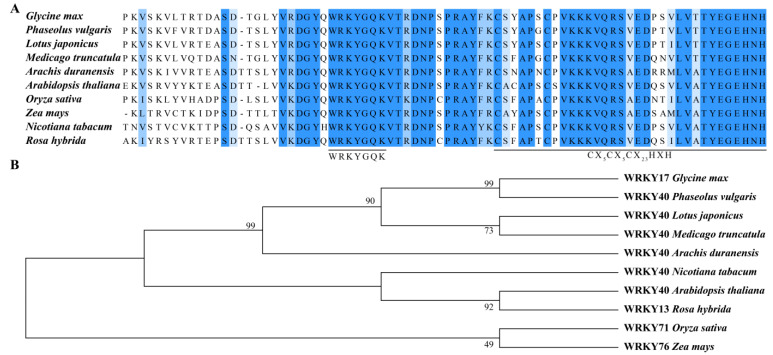
Multiple alignment and phylogenetic relationship of GmWRKY17 with different species. (**A**) Multiple alignments of GmWRKY17 with other WRKY17 proteins from other species. (**B**) Phylogenetic relationship of GmWRKY17 in different species. The number of nodes is the bootstrap value and the number on the branch is the evolutionary distance. Bootstrap replications are 1000.

In order to investigate the conservation of GmWRKY17, a multiple alignment of GmWRKY17 with its leguminous plant homologs in *Phaseolus vulgaris*, *Lotus japonicus*, *Medicago truncatula*, and *Arachis duranensis* showed high similarity to GmWRKY17. The results demonstrated a high degree of conservation at the amino (N) terminus of the WRKY17 proteins across different leguminous plants, with the conserved heptapeptide (WRKYGQK) sequence being completely consistent, reflecting the characteristic features of the WRKY transcription factor (Figure 1A). To further assess the evolutionary relationship of GmWRKY17 with WRKY17 from nine other species, a phylogenetic tree was constructed. The resulting phylogenetic analysis indicated that these WRKY family members form a legume-specific sub-group, and GmWRKY17 shares the highest similarity with WRKY17 from *Phaseolus vulgaris* (>XP_068484043.1) (Figure 1B), which suggests a potential role of *GmWRKY17* in legume nodulation.

### 2.2. Expression Pattern of GmWRKY17 in Response to Rhizobium Inoculation

In this study, complementary DNA (cDNA) synthesized from Wm82 root nodule RNA was utilized as the template for amplifying the full-length *GmWRKY17* CDS using specific primers, resulting in a PCR fragment of approximately 885 base pairs (bp) (Figure S2).

**Figure 2:**
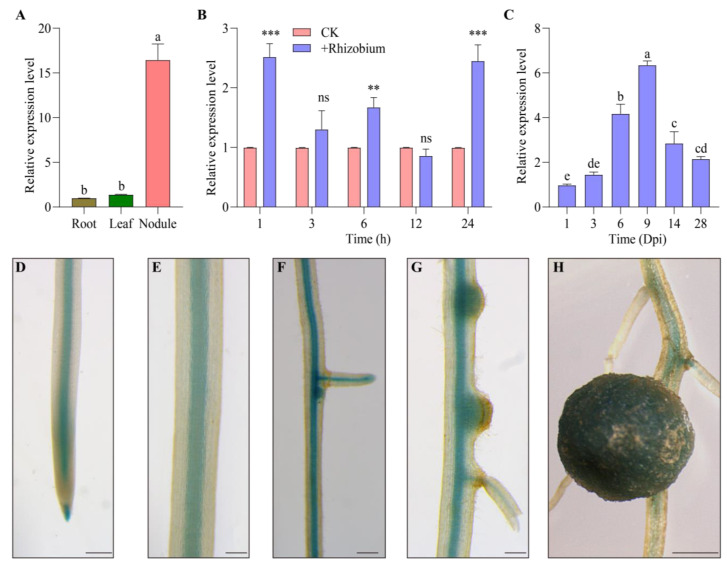
Expression pattern of *GmWRKY17* in soybean nodulation. (**A**) Relative expression levels of *GmWRKY17* in soybean roots, leaves, and nodules at 28 days DAI (Days after inoculation). Tukey Test was used as a post hoc analysis of the ANOVA to confirm significant differences between groups, different groups were assigned different letters (e.g., a and b) to indicate significant relationships between groups, with the same letters indicating no significant differences (*p* > 0.05) and different letters indicating significant differences (*p* < 0.05). (**B**) Expression of *GmWRKY17* in soybean roots at 0, 1, 3, 6, 12, and 24 h after inoculation. ** *p* < 0.01; *** *p* < 0.001; ns, not significant (*p* > 0.05). (**C**) Expression of *GmWRKY17* in soybean roots at 3, 6, 9, 14, and 28 DAI. Tukey Test was used as a post hoc analysis of the ANOVA to confirm significant differences between groups, different groups were assigned different letters (e.g., a, b, c, etc.) to indicate significant relationships between groups, with the same letters indicating no significant differences (*p* > 0.05) and different letters indicating significant differences (*p* < 0.05). (**D**–**H**) Histochemical analysis of *GmWRKY17* expression in transgenic soybean roots and nodules: root tip region (**D**), emerged pericycle (**E**), lateral root primordium (**F**), nodule primordium (**G**), and nodule (**H**). Scale bar in (**D**–**H**) = 5 mm.

In order to investigate the expression pattern of *GmWRKY17*, RT-qPCR was employed in order to assess the transcription levels of *GmWRKY17* in various soybean tissues, as well as inoculated root at different time points. Initially, the relative expression levels of *GmWRKY17* were measured in the roots, leaves, and nodules of soybean at 28 DAI (Days after inoculation). The results demonstrated that *GmWRKY17* exhibited the highest expression levels in the nodules (Figure 2A). In order to examine the temporal expression pattern of *GmWRKY17* in soybean roots in response to rhizobium infection, soybean plants with first trifoliate leaves expanded were inoculated with soybean *Bradyrhizobium (B) diaefficiens* USDA110. The results indicated that *GmWRKY17* exhibited relatively high expression levels during the early stages of inoculation, particularly within the first day after inoculation. While no significant changes in relative expression levels were observed at 3 and 12 h after inoculation, a notable increase was detected at 1, 6, and 24 h after inoculation, suggesting that its expression is induced by the rhizobium infection (Figure 2B). Furthermore, an upward trend in the relative expression levels of *GmWRKY17* was found at 1, 3, 6, and 9 DAI (Figure 2C). Conversely, from 14 to 28 DAI, the expression levels in the roots gradually decreased (Figure 2C). These expression results suggest that *GmWRKY17* may play an important role in different soybean nodulation processes.

In order to further elucidate the tissue expression of *GmWRKY17* during soybean nodulation, the −2 kb promoter region upstream of the *GmWRKY17* ATG start codon was cloned into a vector containing the *β*-glucuronidase (GUS) reporter gene (*proGmWRKY17*:GUS) to generate transgenic soybean hairy roots. Histochemical staining analysis was conducted on the transgenic hairy roots at various developmental stages following rhizobial inoculation. The results indicated that GUS expression was predominantly detected in the root tips, central cylinder sheath, lateral root primordia, and nodule primordia at 3, 5, and 7 DAI (Figure 2D–G). During the process of nodule development, the GUS signal within the root system underwent a gradual attenuation, with a notable expression observed primarily within the nodules at 28 days after inoculation (Figure 2H). These findings suggest that GmWRKY17 may be involved in nodule development and formation. In summary, it is highly likely that GmWRKY17 plays a crucial role in soybean nodulation.

### 2.3. GmWRKY17 Was Localized in the Nucleus

**Figure 3:**
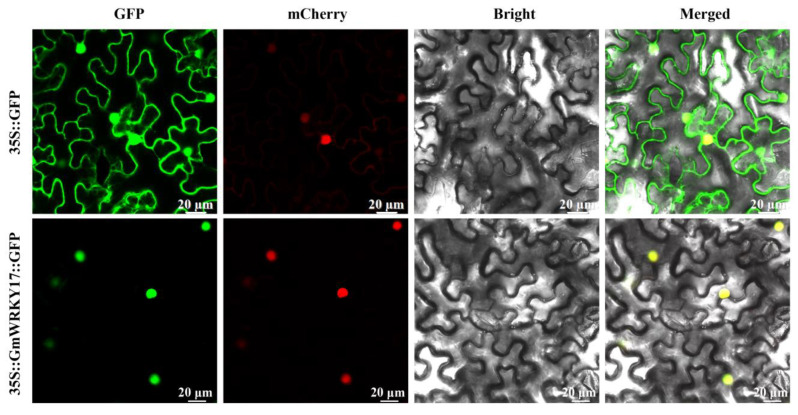
Subcellular localization of GmWRKY17 protein in *N. benthamiana* leaf epidermal cells. Fluorescence of GmWRKY17-GFP was observed in the nucleus of *N. benthamiana* leaf epidermal cells. The 35S-GFP (empty vector) was used as control, localized everywhere in the cell. The 35S-RFP-NLS was used as the nucleus marker. GFP: green fluorescent protein; mCherry: red fluorescent protein (nucleus); Merged: merged image of GFP, mCherry, and bright field. Bars = 20 μm.

It has been reported that WRKY TFs primarily localize in the nucleus to exert their functions. In order to verify whether GmWRKY17 also localizes in the nucleus, a fusion expression vector, 35S::GmWRKY17-GFP, was constructed. The construct was then introduced into Nicotiana benthamiana leaves, and the subcellular localization of the protein was observed using confocal microscopy. The results demonstrated that the 35S::GmWRKY17-GFP fusion protein predominantly resides within the nuclear compartment (Figure 3), implying that GmWRKY17 likely functions as a TF in the nucleus.

### 2.4. GmWRKY17 Is an Important Regulator in Regulating Soybean Nodulation

**Figure 4:**
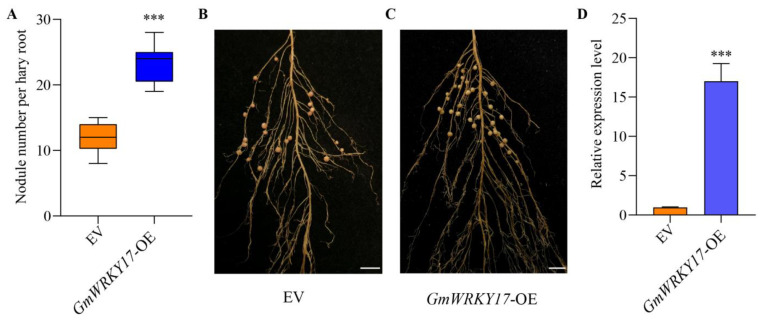
Phenotypic analysis of *GmWRKY17* overexpression. (**A**) Expression level of transgenic hairy roots harboring empty vector and *GmWRKY17*-OE. The expression levels were normalized against the housekeeping gene of soybean *GmCYP2*. Student’s *t*-test was performed (*** *p* < 0.001, *n* = 16). (**B**,**C**) Nodule status of individual transgenic roots expressing EV and *GmWRKY17*-OE at 28 DAI. Bar = 1 cm. EV: empty vector; *GmWRKY17*-OE: overexpression *GmWRKY17*. (**D**) Quantitative analysis of nodule number per hairy root carrying EV and *GmWRKY17*-OE at 28 DPI. Values are the mean ± SD. A total of 16 hairy roots were collected for each biological replicate (*n* = 16, Student’s *t*-test; *** *p* < 0.001).

**Figure 5:**
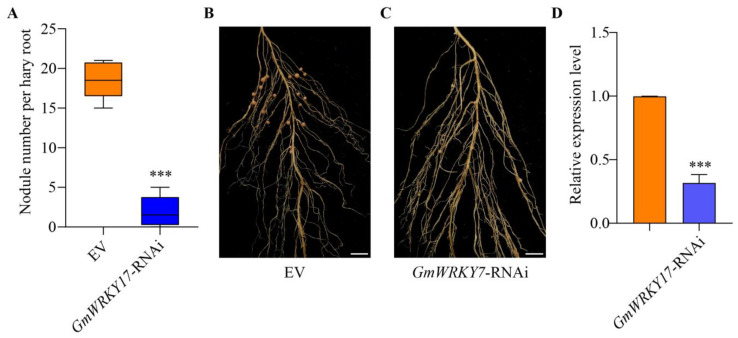
Knocking down *GmWRKY17* inhibits nodulation. (**A**) RT-qPCR analysis of transgenic hairy roots harboring empty vector and *GmWRKY17*-RNAi. The expression levels were normalized against the housekeeping gene of soybean *GmCYP2*. Student’s *t*-test was performed (*** *p* < 0.001, *n* = 16). (**B**,**C**) Nodule status of individual transgenic roots expressing EV and *GmWRKY17*-RNAi at 28 DAI. Bar = 1 cm. EV: empty vector; *GmWRKY17*-RNAi: RNA interference of *GmWRKY17*. (**D**) Quantitative analysis of nodule number per hairy root carrying empty vector and *GmWRKY17*-RNAi at 28 DPI. Values are the mean ± SD. A total of 16 hairy roots were collected for each biological replicate (*n* = 16, Student’s *t*-test; *** *p* < 0.001).

In order to further ascertain the role of GmWRKY17 in soybean nodulation, constructs of transgenic hairy roots expressing empty vector EV), overexpressing *GmWRKY17* (*GmWRKY17*-OE) and *GmWRKY17* RNA interference (*GmWRKY17*-RNAi) were generated. Subsequently, the number of nodules per transformed root was analyzed at 28 DAI. A significant increase in nodule numbers in the *GmWRKY17*-OE roots compared to the controls was observed (Figure 4A–C), suggesting that *GmWRKY17* overexpression promotes soybean nodulation. In the *GmWRKY17*-OE hairy roots, the expression level of *GmWRKY17* increased to approximately 17 times that observed in the *GmWRKY17*-OE roots compared to the empty vector control roots (Figure 4D). Moreover, the silencing of *GmWRKY17* resulted in a substantial reduction in the number of nodules in soybean hairy roots compared to the controls, with the empty vector control averaging approximately 16 nodules per root, while *GmWRKY17*-RNAi roots averaged approximately 2 nodules per root (Figure 5A–C). In the *GmWRKY17*-RNAi hairy roots, the transcription levels of *GmWRKY17* were significantly lower than those of the controls (Figure 5D). These results indicate that silencing of *GmWRKY17* inhibits soybean nodulation. Collectively, these data suggest that *GmWRKY17* may play a positive regulatory role in soybean nodulation.

**Figure 6:**
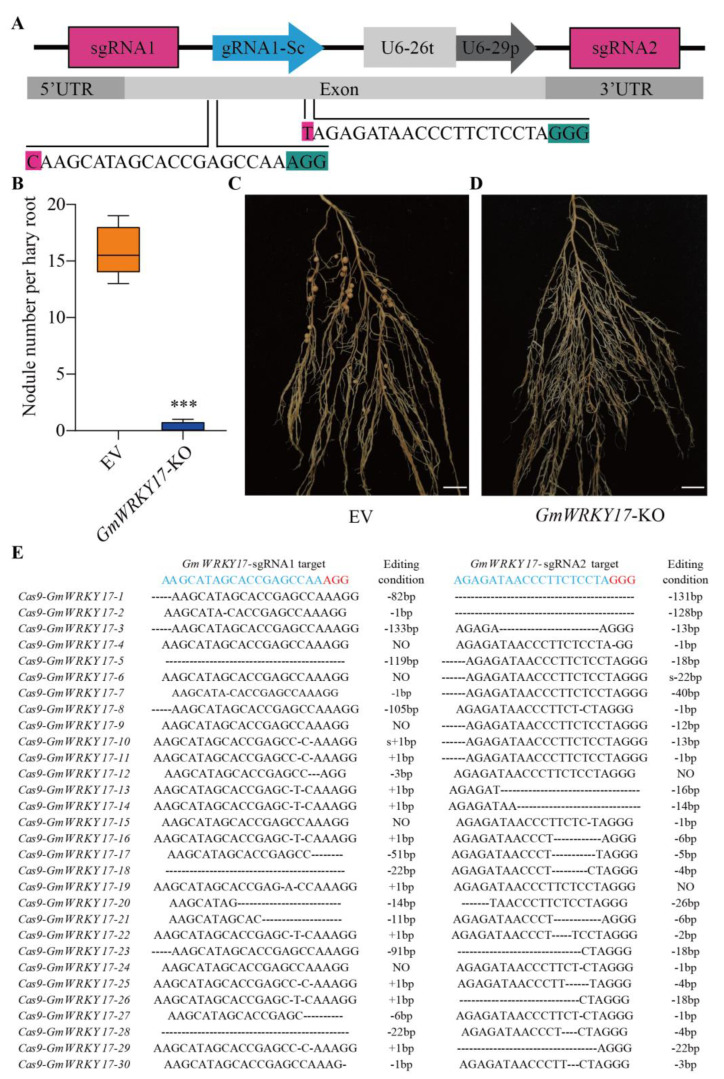
Nodulation phenotypes of *GmWRKY17* knockout mutant hairy roots. (**A**) Schematic diagram of pKSE401-GFP vector construction and two sgRNAs (small guide RNAs) sequences of *GmWRKY17*. (**B**) Quantitative analysis of nodule number per hairy root carrying empty vector and *GmWRKY17*-KO at 28 DAI. Values are the mean ± SD. A total of 24 hairy roots were collected for each biological replicate (*n* = 24, Student’s *t*-test; *** *p* < 0.001). (**C**,**D**) Nodule status of individual transgenic roots expressing empty vector and *GmWRKY17*-KO at 28 DPI. Bar = 1 cm. (**E**) The gene editing conditions in the *GmWRKY17* knockout hairy roots (*n* = 30). Each line in the bars indicates different gene editing conditions in individual hairy roots. The letter “S” stands for base substitution, and “NO” stands for no editing occurs.

In order to provide further validation of the role of GmWRKY17 in soybean nodulation, we employed CRISPR-Cas9 technology to knockout *GmWRKY17*. Two target sites were selected within the coding region of GmWRKY17 and integrated into a binary vector driven by the U6 promoter (Figure 6A). The generation of transgenic hairy roots harboring the *GmWRKY17* knockout (*GmWRKY17*-KO) was then undertaken. The results indicated that *GmWRKY17*-KO led to a significant nodule number reduction (Figure 6B–D). Subsequent to the generation of the *GmWRKY17*-KO hairy roots, genomic DNA was extracted from these roots and utilized as a template for PCR, sequencing, and sequence analyses. These analyses confirmed the knockout of *GmWRKY17* in the transgenic hairy roots (Figure 6E). Collectively, these results indicated that GmWRKY17 functions as a positive regulatory factor in soybean nodulation.

### 2.5. GmWRKY17 Regulates the Expression of Critical Genes Associated with the NF Signaling Pathway

**Figure 7:**
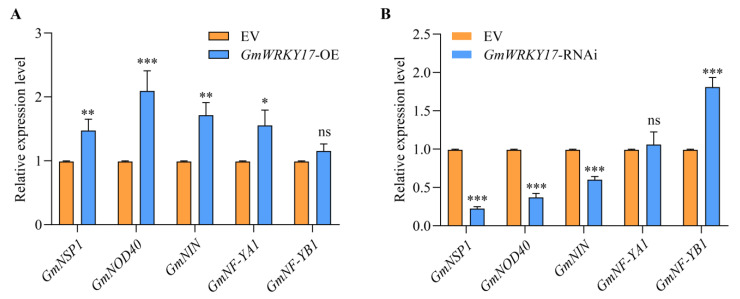
*GmWRKY17* expression alliterating inhibits the transcript levels of nodulation-related genes. (**A**) RT-qPCR analysis of *GmNSP1*, *GmENOD40*, *GmNIN*, *GmNF-YA1*, and *GmNF-YB1* in roots transformed with empty vector and *GmWRKY17*-OE at 28 DAI (*n* = 16). (**B**) RT-qPCR analysis of *GmNSP1*, *GmENOD40*, *GmNIN*, *GmNF-YA1*, and *GmNF-YB1* in roots transformed with empty vector and *GmWRKY17*-RNAi at 28 DPI (*n* = 16). We set the transcript level of the *GmNSP1*, *GmENOD40*, *GmNIN*, *GmNF-YA1*, and *GmNF-YB1* at 28 DAI EV hairy roots as “1”. The transcript amounts in each sample were normalized to those of *GmCYP2* (*n* = 16, Student’s *t*-test; * *p* < 0.05, ** *p* < 0.01, and *** *p* < 0.001; ns, no significance).

Nodule number is primarily regulated by the NF signaling pathway [43]. Given that the *GmWRKY17*-OE significantly increases the number of soybean nodules, whereas the silencing or knockout of *GmWRKY17* leads to a substantial reduction in nodule numbers, it was hypothesized that GmWRKY17 regulates nodulation through the NF signaling pathway. To address this, we examined the relative expression levels of symbiotic nodulation marker genes, including *GmNSP1* [44], *GmENOD40* [45], *GmNIN* [46], *GmNF-YA1*, and *GmNF-YB*1 [47] in the *GmWRKY17* overexpressing and silenced hairy roots of soybean. As demonstrated in Figure 7, compared to the control roots, the relative expression levels of *GmNSP1*, *GmENOD40*, *GmNIN*, and *GmNF-YA1* were significantly increased in the *GmWRKY17*-OE roots (Figure 7A). In contrast, in the *GmWRKY17*-RNAi roots, the relative expression levels of *GmNSP1*, *GmENOD40*, and *GmNIN* were significantly decreased compared to the control roots (Figure 7B), while the relative expression of *GmNF-YB1* was increased in the *GmWRKY17*-RNAi roots (Figure 7B). Consequently, these findings imply that altering the expression of *GmWRKY17* exerts a substantial influence on the expression of genes within the NF signaling pathway. This discovery suggests that GmWRKY17 functions as a regulator in the soybean nodulation process by influencing the expression of NF signaling pathway-associated genes.

## 3. Discussion

The plant hormone signaling regulatory network plays a crucial role in the nodulation regulation system. Among these, auxin and CK promote nodule formation [48,49,50]; while ethylene, JA, ABA, and GA inhibit infection thread formation and nodule development [20]. Additionally, plant hormones exhibit mutual regulatory interactions; for instance, excessive CK and auxin stimulate ethylene biosynthesis, activating plant immune responses, and thereby suppressing nodule formation and development [21]. The promoting effect of CK on nodule formation is supported by substantial genetic evidence. In the absence of rhizobia, the exogenous application of CK or heterologous expression of CK biosynthesis-related genes can induce spontaneous nodulation. Similarly, gain-of-function *lhk*1/*cre1* mutants in barrel clover *Lotus japonicus* and *Medicago truncatula* exhibit spontaneous nodulation phenotypes [51,52]. However, CK levels and distribution in root epidermal cells are strictly regulated, negatively controlling rhizobial infection. Research indicates that CK in root epidermal cells originates from the cortex, and mutations in the CK degradation enzyme CKX3 reduce infection events, suggesting that CK likely acts as negative feedback signals from the cortex to the epidermis to regulate infection [42,51]. Other hormones, such as ABA and GA, also influence nodule formation and bacterial infection. Studies have shown that ABA negatively regulates the early expression of the early nodulin gene *ENOD11* and inhibits NF signaling transduction [41]. However, the crosstalk mechanism of CK and ABA in legume nodulation remains unknown. In this study, we found that GmWRKY17, a homolog of *Rosa hybrida* (Rh)WRKY13, which mediates plant immunity through regulating cytokinin content and abscisic acid signaling, is predominantly expressed in soybean root nodules and functions as a positive regulator in soybean nodulation.

The WRKY TF superfamily, an extensively investigated group of TFs, has attracted considerable research interest due to its diverse biological functions. Since the initial report on WRKY TFs across different species [27], WRKY-type TFs have emerged as key regulators of plant growth and developmental stages, secondary metabolism, responses to environmental stimuli, and pathogen defense [38,44,45,46,47]. In recent years, many investigations into WRKY TFs have employed biotechnology and RNA-Seq methods [48,49]. Most of these studies mainly focused on the whole-genome annotation of WRKY family genes and the expression analyses of select genes involved in responses to abiotic and biotic stresses. However, the elucidation of WRKY TFs’ roles in rhizobium infection responses, as well as their regulatory functions in nodulation and nitrogen fixation, remains a topic requiring further investigation. In this study, based on RT-qPCR and GUS histochemical staining analysis, we found that the *GmWRKY17* exhibits nodule specificity expression and is significantly induced by rhizobium inoculation (Figure 2). We selected GmWRKY17 as the research subject to explore its role in soybean nodulation.

To adapt to nitrogen-deficient soil environments, leguminous plants have evolved a symbiotic nitrogen-fixing relationship with rhizobia to acquire nitrogen from the atmosphere. Research indicates that a moderate increase in the number of nodules can enhance SNF in soybeans and balance the allocation of carbon sources, thereby improving both carbon and nitrogen acquisition, ultimately increasing soybean yield and protein content [53]. Consequently, the nodule number directly influences the nitrogen-fixing capacity of leguminous plants, as well as their growth and yield. Overexpression of *GmWRKY17* in soybean roots resulted in a significantly higher number of nodules in roots compared to control plants (Figure 4), while silencing or knocking out *GmWRKY17* led to a marked decrease in the number of soybean root nodules (Figure 5 and Figure 6). This result demonstrated that WRKY-type transcription factors are involved in the regulation of nodule number in legume.

SNF is an energy-intensive enzyme-catalyzed process [54], and plants employ regulatory mechanisms to modulate nodule number. Numerous genes have been identified that participate in the regulation of nodule quantity. NIN is a crucial transcription factor that governs root nodule organ development and nodule number [26,55]. The expression level of NIN is induced by the inoculation of rhizobia and NF, which requires the involvement of NSP1 and NSP2 [56]. Both NSP1 and NSP2 belong to the GRAS protein family and serve as essential transcriptional regulators for NF-induced signaling [57,58]. *NSP1* is constitutively expressed in the nucleus, which may allow it to be activated by DMI3 within the NF signaling pathway [44]. The NSP2 protein is specifically localized to the nuclear envelope and endoplasm reticulum, accumulating in the nucleus following inoculation with rhizobia or treatment with nodulation factors [59]. Mutation of *NSP1* and *NSP2* fail to induce root hair curling, infection thread formation, and cortical cell division in response to rhizobial inoculation or NF treatment; however, they do not affect root hair tip swelling or branching, nor calcium oscillations [60]. The nuclear factors NF-YA1 and NF-YB1, which are downstream of NIN, play significant roles in cortical cell division and organogenesis of root nodules [47,60]. *ENOD40* is one of the earliest expressed nodulin genes during nodule formation and is critically involved in the formation of nodule primordia during rhizobial infection [45]. *GmWRKY17* is induced by rhizobia, and the overexpression of *GmWRKY17* in transgenic hairy roots significantly increases the relative expression levels of *GmNSP1*, *GmENOD40*, *GmNIN*, and *GmNF-YA1* (Figure 7A). Conversely, the roots of *GmWRKY17*-RNAi exhibit a significant decrease in the relative expression levels of *GmNSP1*, *GmENOD40*, and *GmNIN* (Figure 7B). These findings indicate that GmWRKY17 regulates nodule number by directly modulating or influencing the expression of NF genes, thereby participating in the molecular mechanisms underlying early nodule signaling pathways.

It can be inferred that GmWRKY17 is a multifunctional protein involved in various regulatory pathways. Future research should focus on determining the downstream genes and interaction partners of *GmWRKY17*, thereby revealing its mechanistic role in regulating the nodulation process in soybeans. *GmWRKY17* not only responds to abiotic stress, enhancing the resilience of transgenic lines but also participates in the symbiotic nodulation process between legumes and rhizobia, thereby increasing nodule quantity. This positions *GmWRKY17* as a promising target for molecular breeding strategies aimed at improving legume crops.

## 4. Materials and Methods

### 4.1. Bioinformatics and Phylogenetic Analysis of GmWRKY17

Gene structure mapping of *GmWRKY17* was systematically characterized through the application of GSDS 2.0, an online bioinformatics tool. The secondary structure, phosphorylation, hydrophilicity/hydrophobicity, and conserved domain analyses of the encoded protein were performed utilizing online analysis tools such as ExPASy (http://www.expasy.org/, accessed on 24 February 2025), NetPhos version 3.1, ProtScale (http://web.expasy.org/protscale, accessed on 24 February 2025) from ExPASy, and NCBI blast. The amino acid sequences of WRKY17 from various species were retrieved from the NCBI database. Subsequently, DNAMAN version 9.0 software was employed for multiple sequence alignment of amino acid sequences in different species. A phylogenetic tree was constructed using the Neighbor-Joining method in MEGA 6.0 software, with 1000 bootstrap replicates conducted.

### 4.2. Rhizobial Strain, Plant Materials and Growth Conditions

The rhizobial strain utilized was the *Bradyrhizobium (B.) diaefficiens* strain USDA110. Following the cultivation of the rhizobia to the logarithmic growth phase, they were diluted to a consistent concentration (OD_600_ = 0.08) for inoculation. The soybean variety was *Glycine max* var. Williams 82 (Wm82) [61]. Healthy seeds were selected, surface-sterilized by spraying with 75% ethanol for a few seconds, dried using absorbent paper, and placed in Petri dishes. Subsequently, they were soaked in 1% sodium hypochlorite for 15 min, followed by rinsing 3–4 times with sterile water. The seeds were germinated in sterilized vermiculite within a light incubator. The plants were then transplanted into small black boxes (10 cm × 10 cm × 10 cm) filled with autoclaved vermiculite and grown under a 16 h light (25 °C)/8 h dark (25 °C) cycle in a growth chamber.

### 4.3. RNA Extraction and Real-Time RT-qPCR

High-quality total RNA was successfully purified from distinct plant tissue samples utilizing the RNAiso Easy extraction kit (Takara Biomedical Technology, Beijing, China). The integrity of the RNA and the existence of DNA contamination were assessed through agarose gel electrophoresis. Reverse transcription was carried out using NovoScript^®^ All-in-one RT UltraMix for qPCR (Novoprotein, Beijing, China). Primer Premier 6 software was used for primer design and synthesized by Qingke Biotech (Tsingke Biotechnology Co., Ltd., Beijing, China), with primer information provided in Table S1. Real-time PCR (RT-qPCR) analysis of the *GmWRKY17* was performed on the LineGene 9600 Plus Fluorescence quantitative polymerase chain reaction detection system (BIOER, Hangzhou, China) using the 2 × Q3 SYBR qPCR Master Mix (TOLOBIO Biotechnology, Shanghai, China). Each sample was analyzed in triplicate biological replicates, the internal reference gene was soybean *GmCYP2* [62]. 2^−ΔΔCt^ was used to calculate the relative expression levels of *GmWRKY17* genes [63].

### 4.4. Cloning of the GmWRKY17 Gene and Vector Construction

Using cDNA synthesized from Wm82 nodule RNA as a template, PCR amplification was conducted with a high-fidelity enzyme, The PCR product was subsequently gel-purified and cloned into the pTOPO001-T vector, followed by transformation into Escherichia coli DH5α. Positive clones identified through PCR analysis were sent for sequencing, and alignment using DNAMAN revealed that the open reading frame (ORF) sequence of GmWRKY17 from Wm82 was identical to the reference genome sequence of Wm82 in the Phytozome database. The coding sequence (CDS) of *GmWRKY17* was amplified through the Golden Gate Assembly method and subsequently inserted into the 5′-Xba I and 3′-Kpn I cloning sites of the pUBI-GFP-4*myc vector, thereby constructing the *GmWRKY17*-OE vector. The target sequence of the *GmWRKY17* CDS was incorporated into the pK7GWIW-GFP vector via an LR reaction to generate the *GmWRKY17*-RNAi vector. For the construction of the CRISPR knockout vector, gRNA was designed using the Crispr-P v2.0 software (http://crispr.hzau.edu.cn/CRISPR2/, accessed on 24 February 2025). Two high-scoring sgRNAs were selected and cloned into the pKSE401-GFP vector using pCBC-DT1T2 as a template, resulting in the generation of the *GmWRKY17*-KO vector.

To express the GmWRKY17-GFP fusion protein, the coding region of GmWRKY17 was inserted into the 5′-Hind III and 3′-Sal I cloning sites of the pSuper1300-GFP vector. To construct the *proGmWRKY17*::GUS reporter gene fusion vector, a 2000 bp genomic region prior to the upstream start codon of GmWRKY17 was amplified by PCR and subsequently inserted into the 5′-Hind III and 3′-BamH I cloning sites of the PCAMBIA1391 vector.

### 4.5. Genetic Transformation of Soybean Hairy Roots and Subcellular Localization

In this study, the transient genetic transformation of soybean roots was successfully achieved via the *Agrobacterium rhizogenes* K599 strain [64,65]. Three days post-germination seedlings were utilized for transformation. The K599 solution containing the test plasmid was utilized to infect soybean seedlings at the cut site on the hypocotyl. Subsequently, the soybean seedlings were transferred to pots filled with vermiculite and cultivated in a growth chamber. A LUYOR-3415RG (LUYOR, Shanghai, China) dual-wavelength fluorescent protein excitation light source was used to identify if the transgenic soybean hairy roots were positive. Positive roots were selected for expression detection and nodule number identification. For subcellular localization analysis, leaves from *N. benthamiana* were employed, achieved via transient expression of GFP-tagged vectors. Transformation was carried out using Agrobacterium GV3101, which was injected into the lower epidermis of tobacco leaves. Transformed plants were then cultured under controlled conditions for 24–48 h. Fluorescence signals were observed using a laser confocal scanning microscope (CLSM), and co-localization experiments with specific markers were performed to validate the subcellular localization of the target protein.

### 4.6. Histochemical Analysis of GmWRKY17 Expression

The *proGmWRKY17*:GUS reporter construction was utilized for hairy root transformation. Following inoculation with the *Bradyrhizobium (B.) diaefficiens* strain USDA110, samples were collected at various stages of infection and nodulation for X-Gluc (5-bromo-4-chloro-3-indolyl *β*-D-glucuronide) staining. After overnight incubation at 37 °C, the tissues were washed with ethanol, and GUS activity was observed under an optical microscope.

### 4.7. Statistical Analysis

Data processing and graphical representation were conducted using Microsoft Excel 2010 and GraphPad Prism 8.0. A Student’s *t*-test was performed to generate *p*-values. The statistical differences are marked as follows: *, *p* < 0.05; **, *p* < 0.01; ***, *p* < 0.001.

## 5. Conclusions

In this study, we performed a comprehensive investigation into the structural features of the soybean *GmWRKY17* gene. Further expression profiling revealed that *GmWRKY17* is preferentially expressed in root nodules and exhibits a notable response to rhizobial infection, suggesting its involvement in nodulation-related processes. The function of *GmWRKY17* in nodulation was validated through overexpression, silencing, and CRISPR-Cas9 techniques. Experimental results showed that overexpression of *GmWRKY17* significantly increased nodule number, while silencing and knockout of *GmWRKY17* led to a dramatic reduction in nodulation, suggesting that *GmWRKY17* plays a positive regulatory role in soybean nodulation. RT-qPCR data revealed that variations in *GmWRKY17* expression levels significantly affected the expression of marker genes associated with the NF signaling pathway. Further investigation into the function of *GmWRKY17* in soybean nodulation processes will emphasize its pivotal role as a transcription factor in mediating symbiotic nodulation in legumes.

## Data Availability

Sequence data from this article can be found in the GenBank/EMBL or *Glycine max* Wm82.a4.v1 database.

## Supplementary Materials

The following supporting information can be downloaded at: https://www.mdpi.com/article/10.3390/ijms26072965/s1.
