# Supplementary material for: Molecular Insights into the Positive Role of Soybean Nodulation by GmWRKY17
Source: Int J Mol Sci. 2025 Mar 25;26(7):2965. doi: 10.3390/ijms26072965 (PMC11988455; doi:10.3390/ijms26072965)
Supplement: Supplementary file 1 [file ijms-26-02965-s001.zip › ijms-3543180-supplementary.pdf]

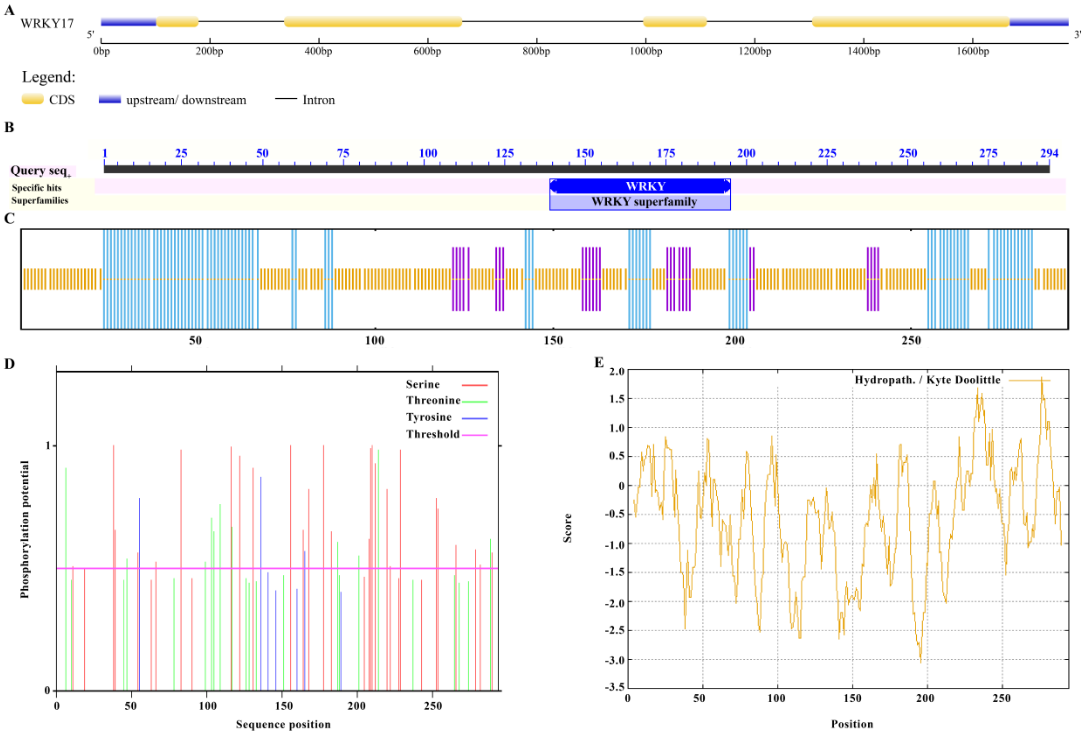

Supplementary Figure S1. Bioinformatics analysis of GmWRKY17. (**A**) Gene structure analysis of *GmWRKY17*. (**B**) Conservative domain prediction of GmWRKY17. (**C**) Secondary structure prediction of GmWRKY17. (**D**) Phosphate site prediction of GmWRKY17. (**E**) Hydropathicity analysis of GmWRKY17.

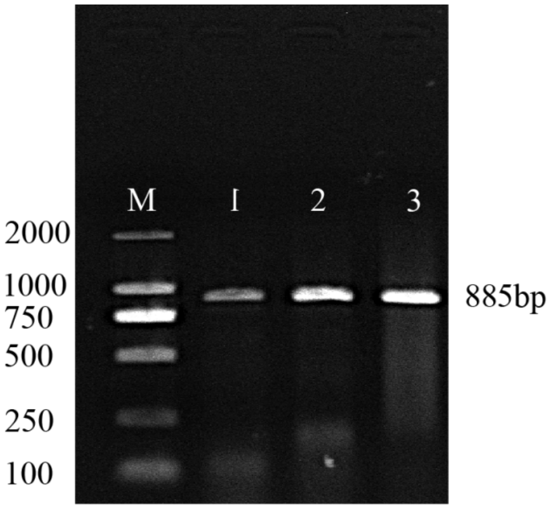

Supplementary Figure S2. Cloning of *GmWRKY17* gene in soybean. M: Trans2K DNA Marker; Lanes 1, 2, and 3 are gene cloning results.

Table S1. Primers designed in this paper.

Name	Sequence
WRKY17-F	ATGGAAATGGAACCGACGTG
WRKY17-R	ACGTTTTCCCACAGAAGTATC
OE-*GmWRKY17*-Forward	TTGATGTGATTACAGTCTAGAATGGAAATGGAACCGACGTG
OE-*GmWRKY17*-Reverse	GTTAATTAACCCGCTGGTACCACGTTTTCCCACAGAAGTATCATCT
GmWRKY17-207-Forward	GGGGACAAGTTTGTACAAAAAAGCAGGCTTCATGGAAATGGAACCGACGTG
GmWRKY17-207-Reverse	GGGGACAAGTTTGTACAAAAAAGCAGGCTTCACGTTTTCCCACAGAAGTATC
Cas9-*GmWRKY17*-BsF	ATATATGGTCTCGATTGAAGCATAGCACCGAGCCAAGTT
Cas9-*GmWRKY17*-F0	TGAAGCATAGCACCGAGCCAAGTTTTAGAGCTAGAAATAGC
Cas9-*GmWRKY17*-R0	AACTAGGAGAAGGGTTATCTCTCAATCTCTTAGTCGACTCTAC
Cas9-*GmWRKY17*-BsR	ATTATTGGTCTCGAAACTAGGAGAAGGGTTATCTCTCAA
qRT-*GmWRKY17*-Forward	GAACTTGTGCAGTTCCGTCG
qRT-*GmWRKY17*-Reverse	TACTGGACAGCTAGGGGCAT
qRT-*GmENOD40*-Forward	TGGACAACACCCTCTAAACCA
qRT-*GmENOD40*-Reverse	GTGAGGGAGTGTGAGGAGTGA
qRT-*GmNIN*-Forward	CATCTTGAGCCTCTACCACC
qRT-*GmNIN*-Reverse	GCTTTGACTCTAAAAGTGCCGG
qRT-*GmNSP1*-Forward	GGTCTATAACTTTTGCTTCCAGC
qRT-*GmNSP*1-Reverse	CAGTGTCTTCGCCAAGAACTTG
qRT-*GmNF-YA1*-Forward	CACGCCATCTACATGCGAC
qRT-*GmNF-YA1*-Reverse	CAGTGTCTTCGCCAAGAACTTG
qRT-*GmNF-YB1*-Forward	GGAGTGCCTTAGGATCTCAACC
qRT-*GmNF-YB1*-Reverse	TACCGCTTGCTTACCGGCTG
qRT-cyp2-Forward	CGGGACCAGTGTGCTTCTTCA
qRT-cyp2-Forward	CCCCTCCACTACAAAGGCTCG
ProGmWRKY17-Forward	CAAGCTTGGCTGCAGGTCGACATCTCTCCAGCCATTCTCACTTG
ProGmWRKY17-Reverse	AATTCCCGGGGATCCGTCGACGAGATATGGAAGGTAGCAGGTGAAA
GmWRKY17-pSuper1300-Forward	ACACGCCAAGCCTCGCTAATGGAAATGGAACCGACGTG
GmWRKY17-pSuper1300-Forward	TCAGGGTCAGCTTGCCGTAACGTTTTCCCACAGAAGTATC
